# Macrophage activation-like syndrome: an immunological entity associated with rapid progression to death in sepsis

**DOI:** 10.1186/s12916-017-0930-5

**Published:** 2017-09-18

**Authors:** Evdoxia Kyriazopoulou, Konstantinos Leventogiannis, Anna Norrby-Teglund, Georgios Dimopoulos, Aikaterini Pantazi, Stylianos E. Orfanos, Nikoletta Rovina, Iraklis Tsangaris, Theologia Gkavogianni, Elektra Botsa, Eleftheria Chassiou, Anastasia Kotanidou, Christina Kontouli, Panagiotis Chaloulis, Dimitrios Velissaris, Athina Savva, Jonas-Sundén Cullberg, Karolina Akinosoglou, Charalambos Gogos, Apostolos Armaganidis, Evangelos J. Giamarellos-Bourboulis, on behalf of the Hellenic Sepsis Study Group

**Affiliations:** 1 4th Department of Internal Medicine, Attikon University Hospital, National and Kapodistrian University of Athens, 1 Rimini Street, 124 62 Athens, Greece; 2 Department for Infectious Diseases and Center for Infectious Medicine, Karolinska Institute, Karolinska University Hospital, Huddinge, Stockholm Sweden; 3 2nd Department of Critical Care Medicine, Attikon University Hospital, National and Kapodistrian University of Athens, Athens, Greece; 4 2nd Department of Internal Medicine, Thriasion Elefsis General Hospital, Elefsina, Greece; 5 1st Department of Pulmonary Medicine, Sotiria Hospital, National and Kapodistrian University of Athens, Athens, Greece; 6 Intensive Care Unit, “G. Gennimatas” General Hospital, Thessaloniki, Greece; 7 1st Department of Critical Care Medicine, Evangelismos Hospital, National and Kapodistrian University of Athens, Athens, Greece; 8 Intensive Care Unit, “Agios Dimitrios” General Hospital, Thessaloniki, Greece; 9 Intensive Care Unit, Theageneion General Hospital, Thessaloniki, Greece; 10 Department of Internal Medicine, University of Patras, Medical School, Patras, Greece

**Keywords:** Macrophages, Sepsis, Ferritin, Interleukin-18, Outcome

## Abstract

**Background:**

A subanalysis of a randomized clinical trial indicated sepsis survival benefit from interleukin (IL)-1 blockade in patients with features of the macrophage activation-like syndrome (MALS). This study aimed to investigate the frequency of MALS and to develop a biomarker of diagnosis and prognosis.

**Methods:**

Patients with infections and systemic inflammatory response syndrome were assigned to one test cohort (*n* = 3417) and a validation cohort (*n* = 1704). MALS was diagnosed for patients scoring positive either for the hemophagocytic syndrome score and/or having both hepatobiliary dysfunction and disseminated intravascular coagulation. Logistic regression analysis was used to estimate the predictive value of MALS for 10-day mortality in both cohorts. Ferritin, sCD163, IL-6, IL-10, IL-18, interferon gamma (IFN-γ), and tumor necrosis factor alpha (TNF-α) were measured in the blood the first 24 h; ferritin measurements were repeated in 747 patients on day 3.

**Results:**

The frequency of MALS was 3.7% and 4.3% in the test and the validation cohort, respectively. In both cohorts, MALS was an independent risk factor for 10-day mortality. A ferritin level above 4420 ng/ml was accompanied by 66.7% and 66% mortality after 28 days, respectively. Ferritin levels above 4420 ng/ml were associated with an increase of IL-6, IL-18, INF-γ, and sCD163 and a decreased IL-10/TNF-α ratio, indicating predominance of pro-inflammatory phenomena. Any less than 15% decrease of ferritin on day 3 was associated with more than 90% sensitivity for unfavorable outcome after 10 days. This high mortality risk was also validated in an independent Swedish cohort (*n* = 109).

**Conclusions:**

MALS is an independent life-threatening entity in sepsis. Ferritin measurements can provide early diagnosis of MALS and may allow for specific treatment.

## Background

Sepsis is defined as a life-threatening organ dysfunction caused by a dysregulated host response to an infection [1]. This definition, suggested a year ago, underlined the role of the host immune response for clinical outcome [1] and challenged the old definition of sepsis as the presence of infection with systemic inflammatory response syndrome (SIRS) [2]. Our current knowledge of the pathophysiology of sepsis suggests that some patients present with hyper-inflammation, some present with immunosupression, and the rest lie somewhere between these two extremes [3]. It is highly plausible that some of the clinical trials conducted between 1994 and 2004 testing diverse strategies of immune intervention failed just because there was no attempt to stratify patients based on the state of immune activation. One of these randomized phase III trials tested the efficacy of anakinra in severe sepsis. Anakinra is a recombinant, non-glycosylated form of the human interleukin (IL)-1 receptor antagonist. Although anakinra did not provide survival benefit for the entire population [4], a subanalysis by Shakoory et al. was recently published focusing on patients with hyper-inflammation [5]. The co-presence of disseminated intravascular coagulation (DIC) and hepatobiliary dysfunction (HBD) was used as an indicator of the predominance of pro-inflammatory phenomena, and this was found in 43 of the 906 patients. Of these 43 patients, 17 were treated with placebo and 26 with anakinra; mortality after 28 days was 65% and 35%, respectively (*p* = 0.0006).

The authors of this post hoc analysis considered that patients with DIC and HBD exhibited features like those of the macrophage activation syndrome (MAS), a state of catastrophic hyper-activation of the innate immune responses. MAS is also known as hemophagocytotic lymphohistiocytosis (HLH) [6]. It is a life-threatening condition of pancytopenia, tissue hemophagocytosis, and early progression to multiple organ dysfunction and death within 10 days [6]. The hallmark of pathogenesis relies on the hyper-activation of tissue macrophages, leading to excessive production of IL-1β, IL-18, and ferritin [7]. Since MAS can also be secondary to malignancies and other autoimmune disorders, a number of criteria have been developed to identify these cases [8, 9]. Presence of bone marrow hemophagocytosis is one of the criteria taken into consideration for the diagnosis of MAS [6, 8, 9]. Since this criterion was investigated neither by Shakoory et al. [5] nor by us in the current study due to the difficulty of performance in every critically ill patient, we prefer to call this entity macrophage activation-like syndrome (MALS). The incidence of MALS in sepsis has never been studied thus far.

The current study aims to (1) describe the presence of MALS in sepsis based on already-published criteria and (2) evaluate serum ferritin as a biomarker for the diagnosis and follow-up of septic patients with MALS.

## Methods

### Study design

The Hellenic Sepsis Study Group has been collecting the data of patients with sepsis since 2006 from 55 study sites across Greece (20 intensive care units and 35 emergency departments (EDs) and departments of internal medicine or surgery). The patients were enrolled after written consent was provided by themselves or by their first degree relatives (for patients unable to consent). The protocol was submitted and accepted from the ethics committees of the participating hospitals. The study involved patients with suspected infection plus at least two SIRS criteria, i.e., sepsis according to the original sepsis definition [2]. Patients infected with the human immunodeficiency virus (HIV) and patients with neutropenia were excluded. Infections and organ dysfunction were defined according to already-published international criteria [10]. From all patients 10 ml blood was collected after peripheral venipuncture within the first 24 h of SIRS presentation, and the procedure was repeated after 48 h. Serum was prepared by centrifugation at 900 g, and samples were transported within the same day to the central lab and stored at – 80 °C until processing.

Clinical data were collected: comorbidities, the Charlson’s comorbidity index, gender, age, medical history, vital signs, complete physical examination including assessment of Glasgow Coma Scale, type of infection, absolute blood cell count, international normalized ratio (INR), activated partial thromboplastin time (aPTT), fibrinogen, fibrinogen degradation products, glucose, urea, creatinine, Na^+^, K^+^, albumin, lactate dehydrogenase, aspartate aminotransferase, alanine aminotransferase, gamma-glutamyl transpeptidase, alkaline phosphatase, bilirubin, arterial pH, partial oxygen pressure, partial carbon dioxide pressure, bicarbonate, lactate, and ratio of partial oxygen pressure to fraction of inspired oxygen (pO_2_/FiO_2_). Blood cultures from peripheral veins and central lines were performed as well as urinalysis, quantitative urine cultures, and quantitative cultures of tracheobronchial secretions, if necessary. Chest X-ray, abdominal ultrasound, and chest and abdominal computed tomography were also performed, if necessary. Acute Physiology and Chronic Health Evaluation (APACHE) II and Sequential Organ Failure Assessment (SOFA) scores as well as data about 28-day outcome were collected.

Commercial enzyme immunosorbent assays were used for the measurements of ferritin (ORGENTEC Diagnostika GmbH, Mainz, Germany), sCD163 (Affymetrix Inc., Santa Clara, CA, USA), tumor necrosis factor (TNF) alpha (R&D Systems, Inc., Minneapolis, MN, USA), IL-6 (Affymetrix Inc.), IL-10 (R&D Systems, Inc.), IL-18 (OriGene Technologies Inc., Rockville, MD, USA), and interferon gamma (IFN-γ) (Affymetrix Inc.). The lower detection limit of ferritin was 5 ng/ml, of sCD163 0.31 ng/ml, and of all cytokines 20 pg/ml. Triglycerides were measured in serum with Lipase/GPO-Trinder (Siemens Healthcare Diagnostics Inc.), and the lower detection limit was 8 mg/dl. All measurements were performed in duplicate and reported by technicians blind to clinical information.

The study endpoints were (1) the frequency of MALS in septic patients using predefined criteria and (2) the development of a biomarker for the early recognition of MALS. For this purpose, the patients were divided into two cohorts, a test cohort and a validation cohort. Patients were randomized into a test cohort and a validation cohort depending on study site and enrollment date in a 2:1 ratio.

### Swedish validation cohort

To demonstrate robustness of findings, it was considered that a cohort from a different geographical region should be used. An independent cohort of 109 severe sepsis/septic shock patients from Sweden was chosen. Characteristics and demographics of this cohort have been previously described [11–14].

### Criteria for MALS and Sepsis-3

The criteria that we used for the classification of MALS were not the same as those suggested by other authors. However, we developed a classification system that could provide equivalent criteria for classification. This is the reason why we classified patients as having MALS and not MAS. More precisely, MALS was diagnosed in every patient who either met the HScore 2014 suggested for adults suffering from autoimmune diseases [15] and/or had both DIC and HBD, as suggested by Shakoory et al. [5]. The HScore, suggested for the diagnosis of hemophagocytosis syndrome (HS), provides specific points for the following variables: immunodeficiency (defined as infection by HIV and/or long-term treatment with immunosuppressive drugs such as cyclosporine, glucocorticoids, and azathioprine; up to 18 points), body temperature (up to 49 points), organomegaly (up to 38 points), cytopenias (up to 34 points), serum ferritin (up to 50 points), triglycerides (up to 64 points), fibrinogen (up to 30 points), aspartate aminotransferase (up to 19 points), and hemophagocytosis in the bone marrow (up to 35 points). The final score varies between 0 and 337 points, so that scores higher than 169 are associated with 90% sensitivity for HS. In the current study, the HScore could range between 0 and 302, since bone marrow aspiration providing 35 points (i.e., 10.4% of the maximal points) was not done routinely in our patients. Taking into consideration that in the original HScore the cutoff value of 169 represented the median of the HScore, it seemed logical to use the median of 151 as the new cutoff value. As a consequence, this modification comprising eight criteria could not be the same as the original HScore, which uses nine criteria. However, it was anticipated to be equivalent to the original HScore. Moreover, we tried to adjust for this limitation in a conservative approach: patients presenting with both HBD and DIC as suggested by Shakoory et al. [5] were also classified as having MALS. HBD was defined by the presence of at least two of the following: (1) serum bilirubin higher than 2.5 mg/dl, (2) aspartate aminotransferase at least two times higher than the upper normal limit, and (3) INR higher than 1.5. DIC was not defined using the definition applied in the manuscript by Shakoory et al. [5]. Instead, in order to achieve better diagnostic sensitivity for DIC, the DIC Score of the International Society of Thrombosis and Hemostasis (ISTH) was used, providing points for the absolute platelet count (maximum 2 points), elevated fibrin-related markers (maximum 3 points), prolonged prothrombin time (maximum 2 points), and fibrinogen level (maximum 1 point). Patients with scores more than or equal to 5 were considered to have overt DIC [16]. All enrolled patients were reclassified into infection and sepsis categories using the new Sepsis-3 definition [17]. Those classified as having sepsis by the new classification and who scored positive for HS and/or HBD/DIC were diagnosed with MALS.

### Statistical analysis

Categorical data were presented as frequencies and confidence intervals (CIs), and quantitative variables as mean ± standard error (SE). Comparisons between groups were done using the Fisher exact test (categorical data) or the Mann-Whitney *U* test (qualitative data). We used logistic regression analysis with odds ratios (ORs) and CIs to investigate if MALS was an independent variable associated with death in the presence of other organ dysfunctions. The receiver operating characteristic (ROC) curve was analyzed for serum ferritin as a biomarker for MALS. Concentrations with specificity higher than 97% were selected. Survival comparisons were done by the log-rank test, and comparisons of the ORs for death by the Tarone and Breslow-Day tests. Non-parametric Spearman correlations were conducted between ferritin and cytokine concentrations. Ferritin concentrations in paired samples were compared with the Wilcoxon test separately for survivors and non-survivors. ROC curve analysis of ferritin changes between days 1 and 3 was done to detect changes with sensitivity greater than 90% for the prognosis of early death. Any *p* value lower than 0.05 was considered as statistically significant.

## Results

**Fig. 1:**
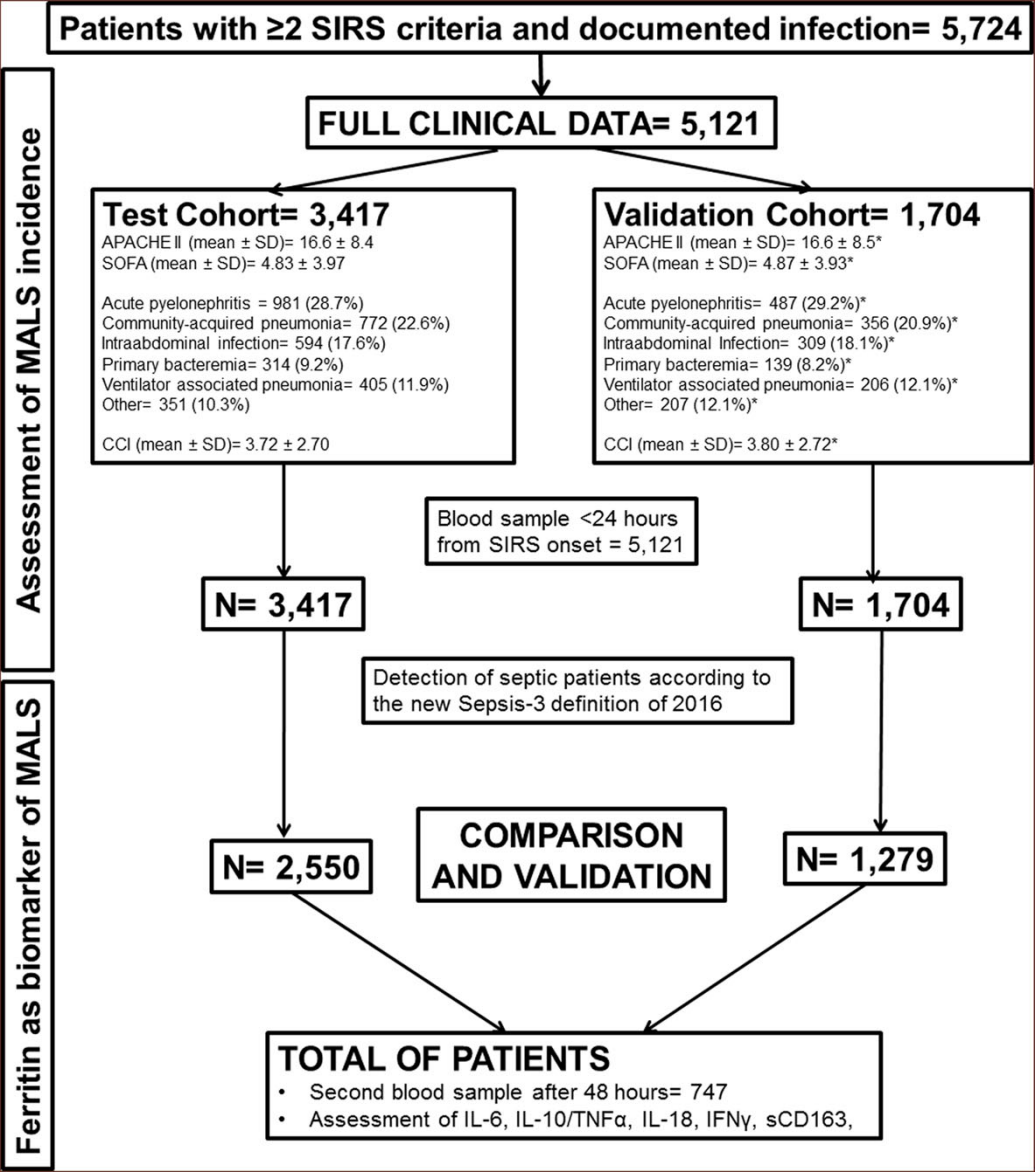
Study flow chart. *APACHE* Acute Physiology and Chronic Health Evaluation, *IFN-γ* interferon gamma, *IL* interleukin, *MALS* macrophage activation-like syndrome, *SD* standard deviation, *SIRS* systemic inflammatory response syndrome, *SOFA* Sequential Organ Failure Assessment, *TNF-α* tumor necrosis factor alpha. *p non-significant between the two cohorts, *CCI*: Charlson's Comorbidity Index

The study flow chart is shown in Fig. 1; 3417 patients were included in the test cohort and 1704 in the validation cohort. Both cohorts were well matched for baseline demographics, SOFA and APACHE II scores, comorbidities, type of infection, and organ dysfunction. As shown in Fig. 1, the analysis was performed in two steps. In the first step, all patients were analyzed, and mortality as well as the MALS characteristics were compared between cohorts. The second step included only patients who met the new Sepsis-3 definitions and aimed to unravel if the levels of serum ferritin could serve as a reliable biomarker of early MALS diagnosis.

**Fig. 2:**
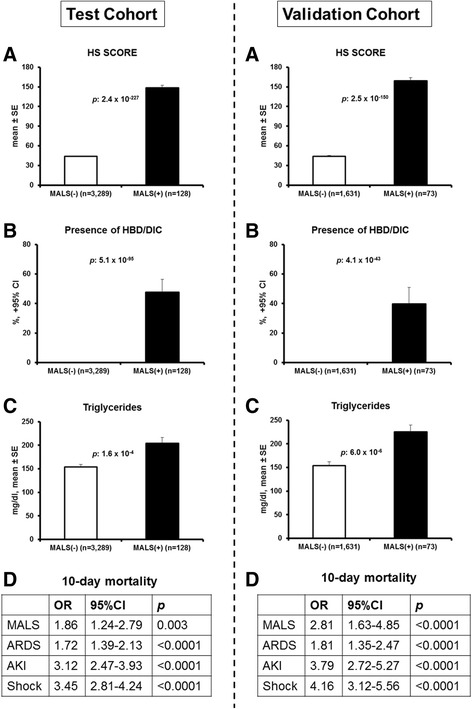
Aspects of macrophage activation-like syndrome (*MALS*) in both cohorts. **a** Score of hemophagocytotic syndrome (*HS*), **b** co-presence of hepatobiliary dysfunction (*HBD*) and disseminated intravascular coagulation (*DIC*), **c** serum triglyceride levels, and **d** logistic regression analysis of factors associated with mortality within 10 days; *p* values are provided. *ARDS* acute respiratory distress syndrome, *AKI* acute kidney injury, *CI* confidence interval, *OR* odds ratio

In the test cohort, 128 (3.7%) patients were classified with MALS. This classification was due to HScore > 151 in 67 patients, to the presence of HBD and DIC in 40 patients, and to the co-presence of HScore > 151 and HBD and DIC in 21 patients. In total, 49 patients had an HScore between 151 and 168, eight of whom also had both HBD and DIC; i.e., 41 patients of the test cohort (1.2%) had a risk of misclassification. Mortality within 10 days was 48.9%, and the presence of MALS was an independent risk factor for unfavorable outcome. In the validation cohort, 73 patients (4.3%; *p* = 0.350 between cohorts) were classified with MALS. This classification was due to HScore > 151 in 44 patients, to the presence of HBD and DIC in 19 patients, and to the co-presence of HScore > 151 and HBD and DIC in 10 patients. In total, 22 patients had an HScore between 151 and 168, four of whom also had both HBD and DIC; i.e., 18 patients of the test cohort (1.1%) had a risk of misclassification. This risk in both cohorts could be well accepted, taking into consideration that the original HScore correctly classified 90% of patients [15]. No differences between the two cohorts were found regarding characteristics of MALS and 10-day mortality (Fig. 2).

**Fig. 3:**
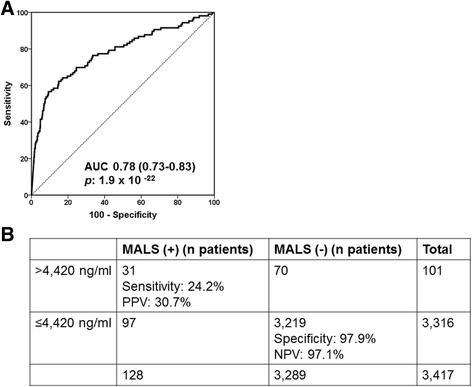
Development of ferritin as biomarker for the detection of macrophage activation-like syndrome (*MALS*) in the test cohort. **a** ROC curve of ferritin for the detection of MALS, *AUC* area under the curve. **b** Sensitivity, specificity, positive predictive value (*PPV*), and negative predictive value (*NPV*) of serum ferritin level 4420 ng/ml for the detection of MALS

**Fig. 4:**
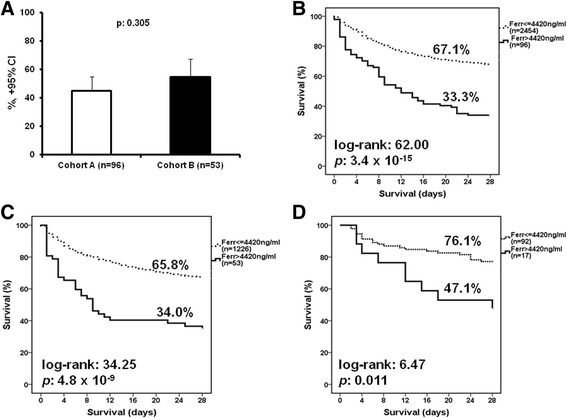
Serum ferritin as biomarker for final outcome in sepsis. **a** Comparison of early mortality between the Greek test cohort and the Greek validation cohort for patients with ferritin > 4420 ng/ml. **b**–**d** Kaplan-Meier curves of survival in the test Greek cohort (**b**), in the Greek validation cohort (**c**), and in the Swedish validation cohort (**d**) for 28 days in relation to ferritin levels. Log-rank tests and *p* values are provided. *Ferr* ferritin

ROC curve analysis (Fig. 3a) performed in the test cohort showed that serum ferritin exceeding 4420 ng/ml was associated with specificity greater than 97% for MALS (Fig. 3b). In the test cohort, 10-day mortality with ferritin above 4420 ng/ml was 44.8% (35.2–54.8%); this value did not differ significantly from that of the validation cohort, where 10-day mortality was 45.2% (36.4–54.3%, *p* = 1.000) (Fig. 4a). The OR for death after 28 days when ferritin was above 4420 ng/ml was 4.07 (95% CI 2.64–6.28) in the test cohort and 3.75 (95% CI 2.10–6.72) in the validation cohort. The ORs did not differ as the *p* value of comparison was 0.827. Survival analysis of the test and validation Greek cohorts and of the Swedish validation cohort ended with considerably higher 28-day mortality among patients with ferritin greater than 4420 ng/ml (Fig. 4b–d).

**Fig. 5:**
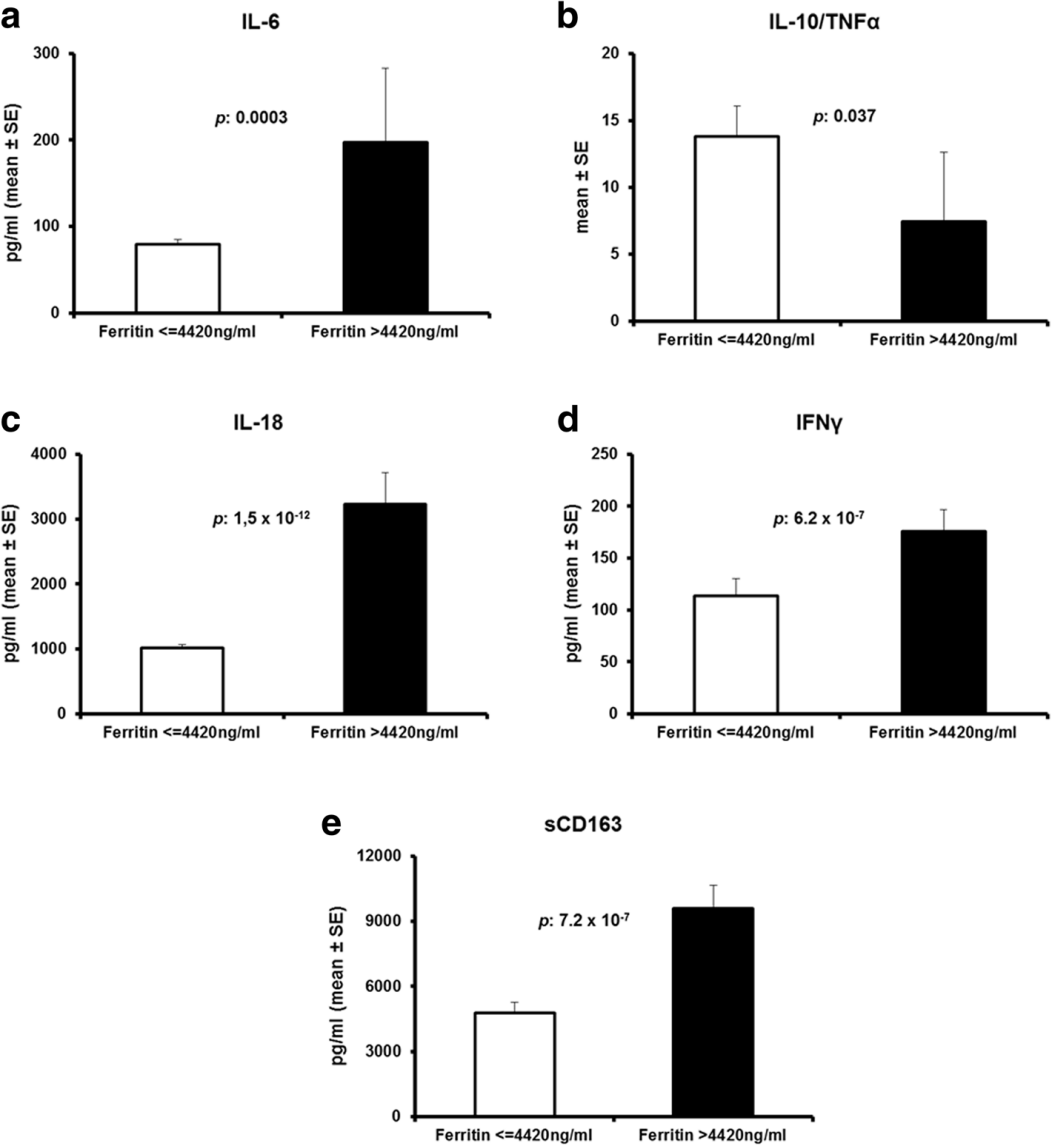
Signs of hyper-inflammation in relation to serum ferritin. Comparison of serum levels of interleukin (IL)-6 (**a**), IL-10/TNF-α ratio (**b**), IL-18 (**c**), interferon gamma (IFN-γ) (**d**), and sCD163 (**e**) in relation to the level of serum ferritin. The ratio IL-10/TNF-α is an expression of the balance between anti-inflammation and hyper-inflammation; *p* values are provided

**Fig. 6:**
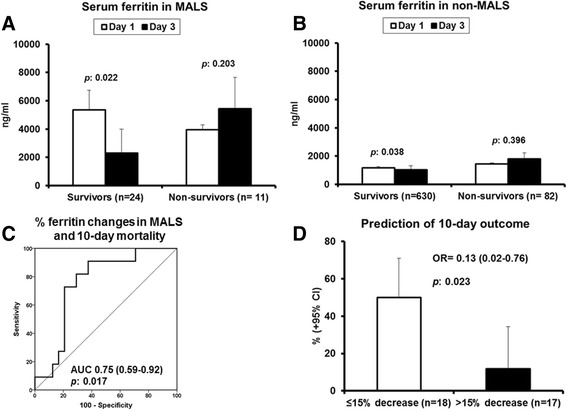
Serum ferritin as a surrogate marker of final outcome among patients with macrophage activation-like syndrome (*MALS*). **a** Serum levels on days 1 and 3 among 10-day survivors and non-survivors of MALS. **b** Serum levels on days 1 and 3 among 10-day survivors and non-survivors without MALS. **c** ROC curve of the % change of serum ferritin between days 1 and 3 as prognostic of death after 10 days among patients with MALS. *AUC* area under the curve. **d** 10-day mortality of patients with MALS with more than or less than 15% ferritin decrease between days 1 and 3. *p* values are provided. *CI* confidence interval, *OR* odds ratio

From the analysis above, we suggest a serum ferritin level above 4420 ng/ml as a potential diagnostic biomarker for MALS. To ensure clinical relevance, such a biomarker for MALS should correlate to hyper-inflammation as well as prognosticate early unfavorable outcome within 10 days. To this end, analysis was performed in patients from both cohorts. IL-6, IL-18, IFN-γ, and sCD163 serum levels were higher and the ratio of serum IL-10/TNF-α was lower for patients with serum ferritin above 4420 ng/ml compared to patients with ferritin ≤ 4420 ng/ml, thus supporting a correlation between elevated ferritin and a pro-inflammatory state (Fig. 5). Positive correlations were found between serum ferritin and serum IL-6 (*r*_s_ = +0.178, *p* = 2.3 × 10^–5^), between serum ferritin and IL-18 (*r*_s_ = +0.267, *p* = 5.3 × 10^–16^), between serum ferritin and IFN-γ (*r*_s_ = +0.250, *p* = 3.0 × 10^–6^), and between serum ferritin and sCD163 (*r*_s_ = +0.340, *p* = 3.0 – × 10^–6^). In 35 patients with MALS, repeat serum ferritin after 48 h was available. Patients who were alive on day 10 demonstrated a significant decrease of serum ferritin, but this was not the case for the non-survivors (Fig. 6a). Similar kinetics of ferritin was not found for patients without MALS (Fig. 6b). ROC curve analysis conducted among patients with MALS showed that changes of ferritin on day 3 were associated with death by day 10. More precisely, coordinate points of the ROC curve showed that any decrease of serum ferritin by day 3 less than 15% predicted early death after 10 days with sensitivity more than 90% (Fig. 6c). Also, the OR for early death was significantly lower for patients with less than 15% decrease of serum ferritin during the first 48 h (Fig. 6d).

## Discussion

The results of the current study underline the presence of MALS as an independent immunological entity associated with unfavorable outcome in sepsis. We found that the frequency of MALS varies between 3.7 and 4.3% in the Greek sepsis cohorts and that it is an independent predictive factor of death within the first 10 days. Our results came from a large-scale test cohort of patients with sepsis and were validated in two cohorts, a Greek and a Swedish one. Our study shows that ferritin serves as a reliable biomarker to exclude MALS, and that levels 4420 ng/ml or above are associated with a diagnostic specificity higher than 97%. Finally, any less than 15% decrease of serum ferritin during the first 48 h was associated with increased chances for unfavorable outcome.

MALS is considered as a hyper-inflammatory response of the host, characterized by deleterious hyper-cytokinemia. It is often triggered by infection, leads to organ dysfunction, and is life-threatening [18]. Since MALS can be a complication not only of an infectious process but also of autoimmune and malignant conditions, a constellation of criteria has been developed for identification of the patients. Although many experts recommend that bone marrow aspiration may be a helpful diagnostic tool [15], the use of this technique is not feasible in daily critical care practice. This limitation is fully recognized in pediatric populations [9, 19]. Although adjustments to the original HScore criteria were made in our study to provide equivalent classification of MALS, the lack of data of bone marrow aspiration is recognized as a limitation.

The parameters used for the diagnosis of HBD and DIC are laboratory tests (aPTT, INR, platelets, fibrinogen, bilirubin, aminotransferases) that are easily and quickly performed even in a setting such as the ED, where patients with sepsis are primarily admitted and resuscitated. On the other side, the aspects of HS, e.g., triglycerides or bone marrow aspiration, are not always available in due course and therefore lead to delay of MALS diagnosis. Such a delay is not acceptable in sepsis, where rapid resuscitation is mandatory. Thus, there is a need for a biomarker for rapid diagnosis, which may be used for personalized care. Our data suggest that serum ferritin is a good candidate biomarker.

Ferritin is increased at levels often exceeding 4000 ng/ml in autoinflammatory syndromes like Still’s disease and acute gouty arthritis [20] that are mediated through excess production of IL-1β and IL-18. Recently, MAS, Still’s disease, septic shock, and catastrophic antiphospholipid syndrome were referred to together as hyperferritinemic syndromes [21]. Although these four conditions are clinically different, they share common features of pathobiology. The term hyperferritinemic syndrome is used because it is suggested that ferritin is not only an expression of hyper-inflammation but also plays a role in enhancing the activation of nuclear factor κB (NF-κB), contributing to further IL-1β production and perpetuation of the inflammation [21].

A reflection of the immune overreaction of the septic host is the elevated circulating IL-18 levels as well as the decreased ratio of IL-10/TNF-α. IL-18, like IL-1β, is synthesized as an inactive pro-peptide in macrophages and dendritic cells which is cleaved by activated caspase-1 into its active moiety. Unlike IL-1β, IL-18 is not consumed through autocrine binding to the cell membrane receptor; hence, high circulating IL-18 is considered an index of elevated production of IL-1β. IL-18 itself enhances the production of IFN-γ, which in turn drives hemophagocytosis, i.e., the hallmark of MAS [22]. Consequently, the increased circulating IL-18 and IFN-γ among patients scoring positive for MALS is likely a direct reflection of the pathobiology. Both cytokines were elevated among our patients with hyperferritinemia further supporting the idea that ferritin levels higher than 4420 ng/ml were an indicator of MALS. The IL-10/TNF-α ratio, which is a marker of the balance between anti-inflammatory and hyper-inflammatory states in sepsis [23], was assessed. Usually a low ratio is compatible with hyper-inflammation. Our analysis showed that this ratio decreased when ferritin exceeded 4420 ng/ml, which was further supportive of high ferritin as a biomarker of hyper-inflammation. It should, however, be emphasized that, in everyday clinical practice, the expression of the major histocompatibility complex II HLA-DR molecule on CD14-circulating monocytes by flow cytometry using fresh blood is an easier way than the IL-10/TNF-α ratio to assess the drive of anti-inflammation. Expression less than 30% is usually indicative of sepsis-induced immunosuppression [24].

During inflammation, macrophages release iron storages and express scavenger receptors like CD163. CD163 is involved in the haptoglobin-hemoglobin complex uptake, and its soluble form can be measured as a protein in serum. Although its role is not clearly understood, sCD163 is considered a reliable marker of macrophage activation [25, 26]. Thus, high levels of sCD163 in our patients with ferritin levels 4420 ng/ml or higher corroborate an activated state of the macrophages in MALS.

In light of the published evidence for sepsis-induced immunosuppression [3], someone may wonder to which state of immune activation a patient with high ferritin and low expression of HLA-DR on circulating monocytes belongs. Our analysis emphasized that a ferritin level above 4420 ng/ml has a very high negative predictive value (NPV) to exclude MALS. To this end, even when a patient presents with traits of sepsis-induced immunosuppression, high concentrations of ferritin above 4420 ng/ml should be considered diagnostic of MALS. This is further emphasized by the association between increase of ferritin and early 10-day mortality that is an intrinsic characteristic of MALS.

Our finding of high ferritin levels as an indicator of MALS appeared to be fully reproducible in an independent cohort of patients with septic shock from Sweden. Although these patients originate from a different healthcare setting, the mortality when ferritin exceeded 4420 ng/ml was seemingly similar to that shown in the large Greek cohorts, showing the robustness of our finding.

## Conclusions

Our results suggest that a serum ferritin level above 4420 ng/ml identifies in a reliable and very specific way the septic patient with MALS. Since the recent subgroup analysis indicated a survival benefit from anakinra treatment among patients with MALS [5], it is plausible that the use of ferritin can identify potential candidates for such a personalized immunotherapy approach in sepsis.

## Abbreviations

APACHE: Acute Physiology and Chronic Health Evaluation
aPTT: Activated partial thromboplastin time
CI: Confidence interval
DIC: Disseminated intravascular coagulation
ED: Emergency department
HBD: Hepatobiliary dysfunction
HLH: Hemophagocytotic lymphohistiocytosis
HS: Hemophagocytosis syndrome
IFN-γ: Interferon gamma
IL: Interleukin
INR: International normalized ratio
ISTH: International Society of Thrombosis and Hemostasis
MALS: Macrophage activation-like syndrome
MAS: Macrophage activation syndrome
OR: Odds ratio
pO_2_/FiO_2_: Ratio of partial oxygen pressure to fraction of inspired oxygen fraction
SOFA: Sequential Organ Failure Assessment
ROC: Receiver operating characteristic
TNF-α: Tumor necrosis factor alpha
